# Neurogenesis Is Reduced at 48 h in the Subventricular Zone Independent of Cell Death in a Piglet Model of Perinatal Hypoxia-Ischemia

**DOI:** 10.3389/fped.2022.793189

**Published:** 2022-04-28

**Authors:** Daniel Alonso-Alconada, Pierre Gressens, Xavier Golay, Nicola J. Robertson

**Affiliations:** ^1^Department of Cell Biology and Histology, School of Medicine and Nursing, University of the Basque Country (UPV/EHU), Leioa, Spain; ^2^NeuroDiderot, Inserm, Université de Paris, Paris, France; ^3^Department of Brain Repair and Rehabilitation, Institute of Neurology, University College London, London, United Kingdom; ^4^Institute for Women’s Health, University College London, London, United Kingdom; ^5^Edinburgh Neuroscience, Centre for Clinical Brain Sciences, The University of Edinburgh, Edinburgh, United Kingdom

**Keywords:** newborn, neonatal brain, hypoxia-ischemia, neurogenesis, subventricular zone

## Abstract

Cellular and tissue damage triggered after hypoxia-ischemia (HI) can be generalized and affect the neurogenic niches present in the central nervous system. As neuroregeneration may be critical for optimizing functional recovery in neonatal encephalopathy, the goal of the present work was to investigate the neurogenic response to HI in the neurogenic niche of the subventricular zone (SVZ) in the neonatal piglet. A total of 13 large white male piglets aged <24 h were randomized into two groups: i) HI group (*n* = 7), animals submitted to transient cerebral HI and resuscitation; and ii) Control group (*n* = 6), non-HI animals. At 48 h, piglets were euthanized, and the SVZ and its surrounding regions, such as caudate and periventricular white matter, were analyzed for histology using hematoxylin-eosin staining and immunohistochemistry by evaluating the presence of cleaved caspase 3 and TUNEL positive cells, together with the cell proliferation/neurogenesis markers Ki67 (cell proliferation), GFAP (neural stem cells processes), Sox2 (neural stem/progenitor cells), and doublecortin (DCX, a marker of immature migrating neuroblasts). Hypoxic-ischemic piglets showed a decrease in cellularity in the SVZ independent of cell death, together with decreased length of neural stem cells processes, neuroblast chains area, DCX immunoreactivity, and lower number of Ki67 + and Ki67 + Sox2 + cells. These data suggest a reduction in both cell proliferation and neurogenesis in the SVZ of the neonatal piglet, which could in turn compromise the replacement of the lost neurons and the achievement of global repair.

## Introduction

During embryonic development, the formation of the central nervous system (CNS) results from a tightly regulated balance between the processes of apoptosis and neurogenesis, which occurs accomplished in time and space (1, 2). At the time of birth, however, the human brain is not yet fully developed [almost 2/3 of the cells are produced after birth; (3)], so intrapartum-related insults like hypoxia-ischemia (HI) may disbalance the apoptosis-neurogenesis equilibrium, obstructing the proper maturational process and leading to life-long sequelae (4). Neonatal HI triggers a series of complex and harmful metabolic cascades that lead to generalized cellular and tissue damage and affect numerous gray and white matter brain regions, but less is known about the effect of HI on the neurogenic niches.

In the CNS, the two neurogenic niches that retain neural stem cells and progenitors with regenerative potential are the subgranular zone of the hippocampal dentate gyrus and the subventricular zone (SVZ) of the lateral ventricle (5). To date, controversy exists around how HI modifies the neurogenic response of the newborn brain. While some reports indicate that HI suppresses the endogenous genesis of neural stem cells and progenitors (6, 7), other studies point out in the opposite direction, suggesting that neurogenesis is increased in the SVZ after HI (8, 9).

Post-injury neurogenesis is a complex process that can be affected by a number of factors, including the duration, type, location, and intensity of damage (10). For instance, the SVZ has shown to be stricken after severe HI (6), whereas in moderate brain injury, neurogenesis was stimulated (9, 11). The period of time since the insult also modulates the neurogenic response to damage: after an initial phase of decreased cell proliferation accompanied by extensive cell death in the rodent SVZ (6, 12), the morphology of the ipsilateral SVZ has shown to increase its size, a phenomenon attributed to augmented cell proliferation (8, 9, 11). Since HI typically occurs at a time when these niches are actively generating new brain cells, we hypothesize that the endogenous neurogenic capability of the SVZ contributing to the plasticity of the newborn brain and/or to tissue remodeling could be compromised if this area is affected.

As described, most of the work on neural stem cells and progenitors of the SVZ has been conducted in rodents. The newborn piglet SVZ shares many anatomical similarities with the SVZ in the human infant, and the SVZ persists beyond fetal development serving as a source of piglet new cells (13). The aim of this study was to investigate if HI affects the SVZ of the neonatal piglet by evaluating its possible changes in cellularity, cell death, cell proliferation, and neurogenesis early after a quantified global cerebral hypoxic-ischemic insult.

## Materials and Methods

All experimentation was in accordance with UK Home Office Guidelines [Animals (Scientific Procedures) Act 1986] and approved by the Animal Care and Use Committee of University College London Biological Services and Institute of Neurology.

### Animal Experiments and Surgical Preparation

Thirteen large white male piglets aged <24 h were included in this study. Briefly, piglets were sedated with intramuscular midazolam (0.2 mg/kg), and arterial O_2_ saturation was monitored (Nonin Medical). Isoflurane anesthesia (4% vol/vol) was applied *via* a facemask during tracheostomy and intubation and was maintained (3% during surgery, 2% otherwise). Piglets were mechanically ventilated to maintain arterial partial pressures of O_2_ (PaO_2_; 8–13 kPa) and CO_2_ (PaCO_2_; 4.5–6.5 kPa) allowing for temperature correction of the arterial blood sample.

An umbilical venous catheter was inserted to infuse maintenance fluids (10% dextrose, 60 ml/kg/day), fentanyl (3–6 μg/kg/h), and antibiotics (benzylpenicillin 50 mg/kg and gentamicin 2.5 mg/kg, every 12 h). An umbilical arterial catheter was inserted for continuous heart rate (HR) and mean arterial blood pressure (MABP) monitoring and 6-h blood sampling to measure PaO_2_, PaCO_2_, pH, electrolytes, glucose (3–10 mmol/L), and lactate (Abbott Laboratories). Bolus infusions of colloid (Gelofusin, B Braun Medical Ltd.) and inotropes maintained MABP >40 mmHg. Arterial lines were maintained by infusing 0.9% saline solution (Baxter, 1 ml/h) with heparin sodium (1 IU/ml) to prevent line blockage. Both common carotid arteries were surgically isolated at the level of the fourth cervical vertebra and encircled by remotely controlled vascular occluders (OC2A, *In Vivo* Metric). After surgery, piglets were positioned prone in a plastic pod with their heads immobilized.

### Cerebral Hypoxia-Ischemia

Before insult, piglets were randomized into two groups, using a computer-generated randomization sequence and opaque sequentially numbered envelopes. The hypoxic-ischemic (HI, *n* = 7) insult was performed according to the original hypoxia-ischemia protocol with transient carotid artery occlusion and contemporaneous hypoxia (14–16). Non-HI animals were not positioned in the scanner nor were subjected to hypoxia or ischemia, serving as controls (Control, *n* = 6).

A magnetic resonance spectroscopy (MRS) surface coil was secured to the cranium, and the animal was positioned in a 9.4 Tesla Agilent MRI scanner, while in the MRI scanner, transient HI was induced by remote occlusion of both common carotid arteries, using inflatable vascular occluders, and reducing the fraction of inspired oxygen (FiO_2_) to 12% (vol/vol).

During HI (*n* = 7), cerebral energetics were monitored every 2 min by phosphorus (31P) MRS, and the β-nucleotide triphosphate (β-NTP; mainly ATP) peak height was automatically measured. When β-NTP peak height had fallen to 40% of baseline, FiO_2_ was adjusted in order to stabilize β-NTP at that level for 12.5 min. At the end of this 12.5-min period, the occluders were deflated and FiO_2_ was normalized; 31P spectra were acquired for a further 1 h to monitor recovery from HI. The time integral of the decrement of β-NTP/EPP [EPP = exchangeable phosphate pool = inorganic phosphate + phosphocreatine + (2γ + β)-NTP] during HI and the first 1 h of resuscitation quantified the acute energy depletion. Piglets were maintained normothermic (rectal temperature 38.5°C) throughout the entire experiment by using a warmed water mattress (Tecotherm) above and below the animal. All animals received continuous physiological monitoring (SA instruments) and intensive life support throughout experimentation.

### Histology and Immunohistochemistry

At 48 h, piglets were euthanized with pentobarbital, and the brain was fixed by cardiac perfusion with cold 4% paraformaldehyde, dissected out, and post-fixed at 4°C in 2% paraformaldehyde for 7 days; 5-mm-thick coronal slices of the right hemisphere, starting from anterior to the optic chiasma, were embedded in paraffin, sectioned to 5 μm thickness, and stained with H&E to validate the bregma for analysis.

**FIGURE 1:**
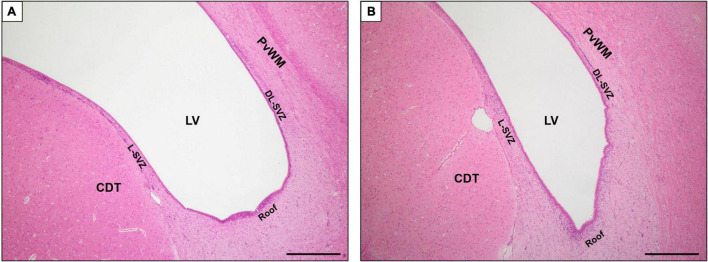
Magnified image of coronal section of the SVZ at bregma –2.0 **(A)** and –4.0 **(B)** brain levels sampled in this study. H&E staining. LV, lateral ventricle; SVZ, subventricular zone; DL-SVZ, dorsolateral-SVZ; L-SVZ, lateral-SVZ. Original magnification 40×. Scale bar: 500 μm.

For each animal, 2 levels (bregma −2.0 and −4.0) were evaluated. There were five regions of interest, namely, the SVZ, which was in turn divided into 3 subareas (Figure 1): roof, dorsolateral-SVZ (DL-SVZ, close to the periventricular white matter), and lateral-SVZ (L-SVZ, apposed to the caudate nucleus); and the caudate nucleus and the periventricular white matter, both being SVZ-surrounding regions (Figure 1).

### Hematoxylin-Eosin Staining

Paraffin-embedded brain samples were stained following the automated procedure corresponding to Hematoxylin-Eosin (H&E) staining by a Shandon Varistain V24-4 (Thermo Electron Corporation, United States) using Harris hematoxylin (Shandon Gill 2 Hematoxylin, Thermo Scientific, United States) and eosin Y (Shandon Eosin-Y Alcoholic, Thermo Scientific, United States).

### Histological Examination of Cell Death in Hematoxylin-Eosin Samples

Histological changes were evaluated in the SVZ (roof, DL-SVZ, and L-SVZ), caudate nucleus, and periventricular white matter brain regions at both bregma −2.0 and −4.0 levels (Supplementary Figures 1, 2) by using H&E staining and analyzed with Fiji/ImageJ image software. For each animal, level, and section, a total of fifteen non-overlapping microphotographs (3 from each area) were taken at 400 × magnification in a light field optical microscope (Olympus BX50F4, Japan). A blinded histologist counted morphologically well-preserved cells (undamaged), together with cells with apoptotic or necrotic features. Apoptotic-like cells were characterized by the presence of nuclear karyorrhexis and low cytoplasmic change, whereas necrotic cells were identified by a pyknotic nucleus or no nucleus, along with a swollen, eosinophilic cytoplasm (17). We did not count apoptotic nor necrotic profiles that were within or adjacent to blood vessels to avoid including apoptotic/necrotic endothelial and white blood cells. The undamaged, apoptotic and necrotic cell count was averaged from 3 high-power fields from 3 slides from the same region in each animal, and values are given as cells per mm^2^.

### Transferase-Mediated Incorporation of Digoxigenin-Labeled Nucleotide

DNA fragmentation was revealed by using the terminal deoxynucleotidyl transferase-mediated incorporation of digoxigenin-labeled nucleotide (TUNEL) assay (Roche, Burgess Hill, United Kingdom). Briefly, *in situ* end-labeling of fragmented DNA was carried out on brain slices that were first deparaffinated, hydrated, pretreated in 3% H_2_O_2_, and subjected to a protease-K digestion (Promega, Southampton, United Kingdom). TUNEL was visualized using avidin-biotinylated horseradish complex (ABC, Vector Laboratories, Peterborough, United Kingdom) and diaminobenzidine/H_2_O_2_ (DAB, Sigma, Poole, United Kingdom) enhanced with CoSO_4_ and NiCl_2_. Finally, TUNEL sections were dehydrated and cover-slipped with DPX (VWR, Leighton Buzzard, United Kingdom).

### Caspase 3 Immunohistochemistry

After deparaffination, antigen retrieval was performed using a pH 6 solution of 10 mM sodium citrate + 0.05% Tween20 in distilled water where samples were boiled 3 times before being kept for 20 min at 95–98°C. After cooling at room temperature, the endogenous peroxidase was blocked, and samples were incubated in 5% bovine serum albumin blocking buffer. Brain slices were then incubated with primary antibody rabbit anti-Caspase 3 (1:100, 9661 L, Cell Signaling, United States) overnight. The next day, samples were incubated with a biotin-conjugated secondary antibody (1:500, goat anti-rabbit, 65-6140, Invitrogen, United States) for 1 h at room temperature followed by horseradish peroxidase-streptavidin conjugate (1:500, 43-4323, Thermo Fisher, United States) plus diaminobenzidine. Right after, sections were counterstained with hematoxylin and mounted with DPX.

An investigator blind to the treatment group performed the quantitative analyses of caspase 3 expression. For each level, section, and brain region, caspase-3 positive cells were counted in three fields (at 40 × magnification, with an area of 0.077 mm^2^) and the average was converted into counts per mm^2^.

### Fluorescent Immunohistochemistry

Brain slices for fluorescence immunohistochemistry were managed as detailed in the “Immunohistochemistry” section until incubation with primary antibody. For single labeling, radial-glia/neural stem cells were identified using an anti-glial fibrillary acidic protein (GFAP) antibody to assess neurogenic activity close to the ventricular wall (mouse anti-GFAP, 1:100, MA5-12023, Thermo Fisher, United States); an anti-doublecortin (DCX) antibody was used to identify young neurons/neuroblasts (mouse anti-DCX, 1:50, sc-271390, Santa Cruz Biotechnology, United States); cell proliferation was identified by an anti-Ki67 antibody (mouse anti-Ki67; 1:50, STJ96966, St Johns Labs, United Kingdom). Double immunohistochemical staining was used to detect the cellular co-localization of Ki67 with Sox2 (neural stem/progenitor cells; 1:100, Santa Cruz Biotechnology, United States). Immunoreactivity was revealed using Alexa Fluor 488 and Texas Red (1:300, Thermo Fisher, United States) secondary antibodies incubated in the dark for 1 h at room temperature. After final washes, fluoromount aqueous mounting medium (F4680, Sigma) was added, and each section was covered by a cover slip. Negative controls received identical treatment except for the omission of primary antibodies and showed no specific staining.

### Histological Evaluation of Markers of Neurogenesis

Sections were examined using Fiji/ImageJ image software, and analyses and quantifications were performed by two independent investigators blinded to the treatment group. We extended the analysis including the cell-dense band of DCX + young neurons along the walls of the lateral ventricle levels (Supplementary Figure 3). The SVZ is further subdivided into roof, dorsolateral-SVZ (DL-SVZ, close to the periventricular white matter), and lateral-SVZ (L-SVZ, apposed to the caudate nucleus). For each animal, level, and section, a total of nine non-overlapping microphotographs (3 from each area) were taken at 200 × or 400 × magnification.

The length of the GFAP positive cells processes lining the SVZ was measured and averaged using Fiji/ImageJ software in three separate microscopic fields at 40 × in the roof, DL-SVZ, and L-SVZ areas with a fluorescence laser microscope. The software was previously calibrated, and the mean length of the processes was obtained after 6 random measurements (Supplementary Figure 4) for each photograph (18). Values are given as μm.

Quantification of Ki67 + and Sox2 + Ki67 + cells was performed in three non-adjacent fields of view at 20 × magnification along the DL-SVZ and L-SVZ edges of the lateral ventricle (18, 19). In each case, the mean of Ki67 + and Sox2 + Ki67 + cells were divided by the area to obtain a measurement of cells per mm^2^.

From sections stained with H&E, photomicrographs were obtained, and the area of neuroblast chains in the SVZ [high density of cells that take up hematoxylin that corresponds to DCX, (20)] was traced and measured in square millimeters.

For DCX fluorescent immunohistochemical evaluation, all the immuno-positive areas along the SVZ were digitalized, and whole fluorescence was obtained because of the confluence of the expression (21). Neuroblasts generated from type 2 progenitors express markers of the neuronal lineage like DCX from their first expression of neuronal lineage and during migration, well before they mature as neurons, so DCX thus is a good early marker in human fetal brain and also in animal studies of neural cell lineage in neurepithelium (22).

### Statistical Analysis

A two-tailed, unpaired Student’s *t*-test was performed for comparisons; data were considered significantly different if *p* < 0.05. Bar graphs appear as mean with 95%CI. Statistical analysis was performed using the Graphpad Prism 8 software package (GraphPad Software, Inc., La Jolla, CA, United States).

## Results

### Hypoxia-Ischemia Induced a Decrease in Cellularity in the Neonatal Piglet Subventricular Zone, Caudate Nucleus, and Periventricular White Matter

In the sagittal section, the SVZ can be easily identified using conventional histological stains as an aggregation of small darkly stained cells located immediately adjacent to the ventricles and descending along the length of the dorsolateral (DL, close to the periventricular white matter) and lateral (L-SVZ, close to the caudate nucleus) walls of the lateral ventricle (Figure 1).

**FIGURE 2:**
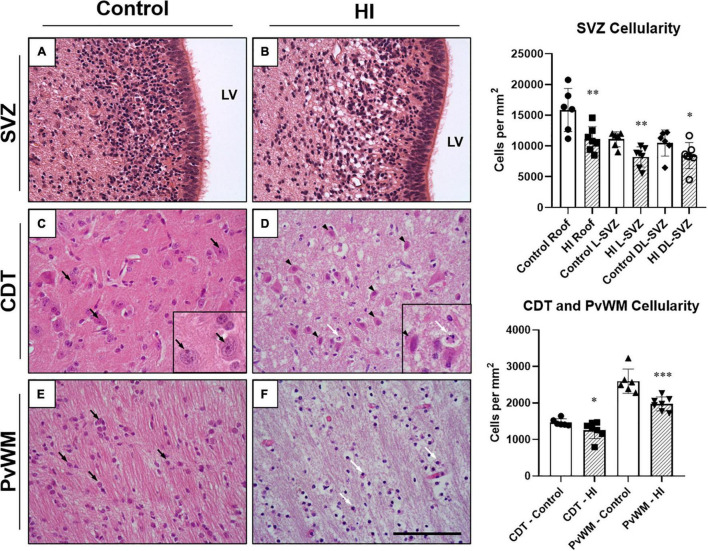
Representative microphotographs of the subventricular zone (SVZ), caudate nucleus (CDT), and periventricular white matter (PvWM) brain regions from control and HI piglets. The piglet SVZ is a highly cellular area with small and closely situated to each other round cells **(A)**. SVZ appeared swollen at 48 h after HI but with very less or absent necrotic or apoptotic features **(B)**. In CDT, well-demarcated cells (black arrows) with well-defined nucleolus (seen in the inset at 630 × magnification), and dense neuropil were observed in control piglets **(C)**. In contrast, in HI animals **(D)** cells with necrotic (black arrowheads) and apoptotic (white arrow) features were present (seen in the inset at 630 × magnification), with diffuse edematous matrix and hypodense neuropil. In PvWM, black arrows identify examples of morphologically undamaged oligodendrocytes **(E)**. **(F)** Shows damaged cells (white arrows) together with diffuse edema and disrupted neuropil from an HI-injured piglet. Graphs: HI reduced the cellularity of the roof (**p < 0.01), L-SVZ (**p < 0.01), and DL-SVZ (*p < 0.05) subareas of the SVZ, an effect also observed in CDT (*p < 0.05) and PvWM (***p < 0.001) cell counts. LV, lateral ventricle; SVZ, Subventricular zone; CDT, caudate nucleus; PvWM, periventricular white matter. H&E staining. Original magnification 400×. Scale bar: 100 μm.

We first evaluated the cellularity of the SVZ, caudate nucleus, and periventricular white matter quantifying the number of morphologically well-preserved (undamaged) cells. After a HI event, the cellularity of the SVZ of the neonatal piglet was reduced. The roof (*p* < 0.01), the L-SVZ (*p* < 0.01), and the DL-SVZ (*p* < 0.05) subareas showed significantly lower counts of morphologically well-preserved cells after neonatal HI (Figure 2, upper graph). Quantitative evaluation of the caudate nucleus (*p* < 0.05) and periventricular white matter’s cellularity (*p* < 0.001) confirmed the decrease in well-preserved cells in the asphyctic piglet in both SVZ-surrounding regions (Figure 2, lower graph).

### The Neonatal Piglet Subventricular Zone Is Resistant to Hypoxia-Ischemia-Induced Cell Death

**FIGURE 3:**
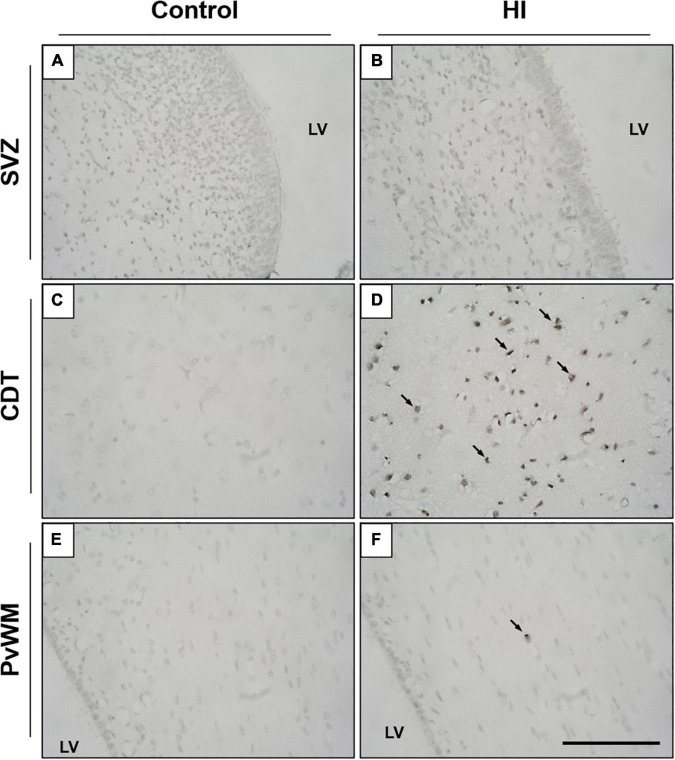
Representative microphotographs of TUNEL immunostaining in the SVZ, caudate nucleus (CDT), and periventricular white matter (PvWM) brain regions from control **(A,C,E)** and HI **(B,D,F)** piglets. SVZ from both experimental groups showed no signs of cell death, as no TUNEL positive nuclei were observed **(A,B)**. In CDT and PvWM, however, immunoreactive cells are observed after HI, especially in the former [black arrows, **(D)**]. LV, lateral ventricle; SVZ, subventricular zone; CDT, caudate nucleus; PvWM, periventricular white matter. Original magnification 400×. Scale bar, 100 μm.

Although the SVZ appeared swollen at 48 h of recovery from HI, H&E-stained samples revealed less death in this area, suggesting that these cells are relatively resistant to damage. A quantitative evaluation later revealed that damaged cells (either with necrotic or apoptotic features) were rarely observed in the SVZ of control (Figure 2A) or HI group (Figure 2B): values were very low for necrotic- (0–0.8 cells per mm^2^) or apoptotic-like cells (0.7–1.2 cells per mm^2^) for both experimental groups, with no differences between control and HI in the three SVZ subareas evaluated (roof, DL-SVZ, or L-SVZ). The absence of cell death in the SVZ was further confirmed when studying TUNEL immune-stained slices from both control and HI piglets (Figures 3A,B).

Later, we focused on the caudate nucleus and periventricular white matter. In H&E-stained samples, we observed extensive cell death in both the caudate nucleus (Figure 2D) and periventricular white matter (Figure 2F) after HI. Caudate nucleus and periventricular white matter of HI piglets showed increased perivascular and pericellular space suggesting edema. Additional changes in the surrounding neuropil included swelling of endothelial cells. Damaged cells appeared necrotic in appearance (nuclear pyknosis with a swollen, eosinophilic cytoplasm) and also apoptotic (fragmented, rounded, dense chromatin with minimal cytoplasmic change) (Figures 2D,F). Quantification of characteristic morphologically altered cell nuclei and cytoplasm revealed notorious necrotic and apoptotic processes in the caudate nucleus and periventricular white matter after HI: caudate nucleus showed high counts of necrotic (Control:2.3 ± 12.6 vs. HI: 99.31 ± 77.4 cells per mm^2^; *p* < 0.01) and apoptotic (Control:1.9 ± 11.2 vs. HI: 6.8 ± 11.0 cells per mm^2^; *p* < 0.002) cell death, and an increase was observed for periventricular white matter for necrosis (2.9 ± 11.4 vs. 99.5 ± 52.2 cells per mm^2^; *p* < 0.05) or apoptosis (2.6 ± 10.4 vs. 1.2 ± 3.5 cells per mm^2^; *p* < 0.05). The lack of DNA fragmentation described above for the SVZ (Figures 3C,E) contrasted with the obvious presence in the caudate nucleus and periventricular white matter after HI (Figures 3D,F) using TUNEL immunohistochemistry.

### Caspase 3 Expression in the Subventricular Zone Is Maintained After Hypoxia-Ischemia

**FIGURE 4:**
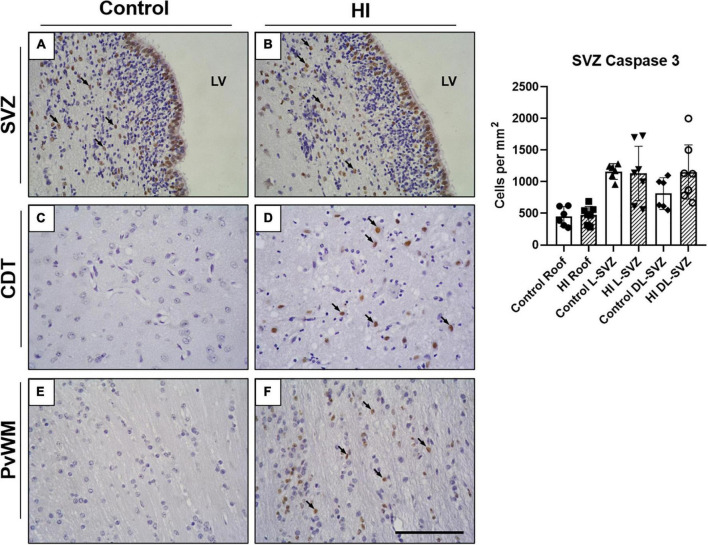
Representative microphotographs of Caspase-3 immunostaining in SVZ, caudate nucleus (CDT), and periventricular white matter (PvWM) brain regions from control **(A,C,E)** and HI **(B,D,F)** piglets. Caspase 3 positive cells can be observed in the SVZ of both experimental groups (black arrows), including the ependymal layer and the underneath SVZ **(A,B)**. Caspase-3 immunoreactivity was also evident in CDT **(D)** and PvWM **(F)** from HI animals. Graph: no differences were observed in caspase-3 positive cell counts in the roof, L-SVZ, or DL-SVZ subareas of the SVZ between control and HI piglets. LV, lateral ventricle; SVZ, subventricular zone; CDT, caudate nucleus; PvWM, periventricular white matter. Original magnification 400×. Scale bar: 100 μm.

Immunohistochemical staining showed that caspase-3 was present in the SVZ of both control and HI animals (Figure 4). In the ependymal layer, most of the cells appear positively stained. Ependymal cells can be distinguished by their location, larger nuclei, ciliated apical domain, and organization in a simple epithelium. These cells were not included in the counts.

Caspase-3 + cells were also observed in the zone immediately subjacent to the ependymal layer of control and HI piglets (Figures 4A,B), but the quantitative analysis did not reveal differences in the counts per mm^2^ in the three SVZ subareas (Figure 4, graph). Further, caspase-3 + nuclei were from non-pyknotic cells. Caudate nucleus and periventricular white matter from control piglets showed a low presence of caspase-3, contrasting with extensively labeled HI samples.

### Hypoxia-Ischemia Reduced the Length of Glial Fibrillary Acidic Protein + Cells Processes

**FIGURE 5:**
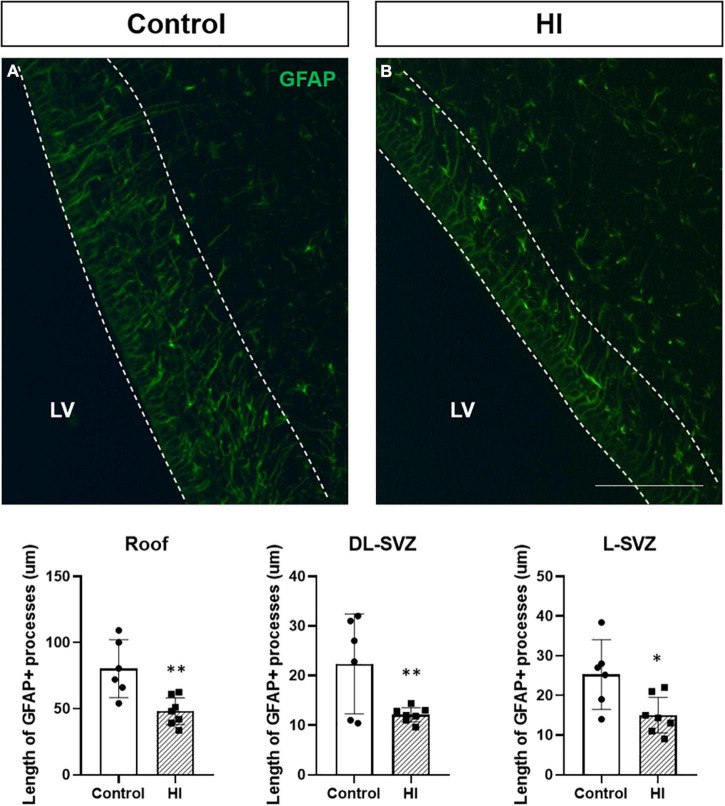
Fluorescent microphotographs of GFAP immune-stained samples from control and HI piglets. GFAP reveals the length of the processes of radial-glia/neural stem cells in the SVZ **(A)**. HI [**(B)** and graph] significantly reduced the length of GFAP + processes in the three subareas of the SVZ: roof (^**^p < 0.01), DL-SVZ (^**^p < 0.01), and L-SVZ (*p < 0.05). LV, lateral ventricle; SVZ, subventricular zone; CDT, caudate nucleus; PvWM, periventricular white matter. Original magnification 400×. Scale bar: 100 μm.

The process length of GFAP + cells (radial-glia like cells or type 1 or neural stem cells) in the SVZ was measured in the three SVZ subareas (i.e., roof, DL-SVZ, and L-SVZ) to assess the neurogenic activity close to the ventricular wall. GFAP + cells showed a large cytoplasm with thin processes that can extend several micrometers away from the ependyma lining the lateral ventricle toward the inner brain parenchyma (Figure 5). The length of GFAP + processes differed from each SVZ subarea being longer in the roof and decreasing its size when getting away from this area along the DL- and L-SVZs. When comparing control and HI groups, HI significantly (*p* < 0.01 for roof and DL-SVZ; *p* < 0.05 for L-SVZ) reduced the length of GFAP + processes in the three subareas of the SVZ (Figure 5, graph).

### Hypoxia-Ischemia Diminished Cell Proliferation in the Subventricular Zone

**FIGURE 6:**
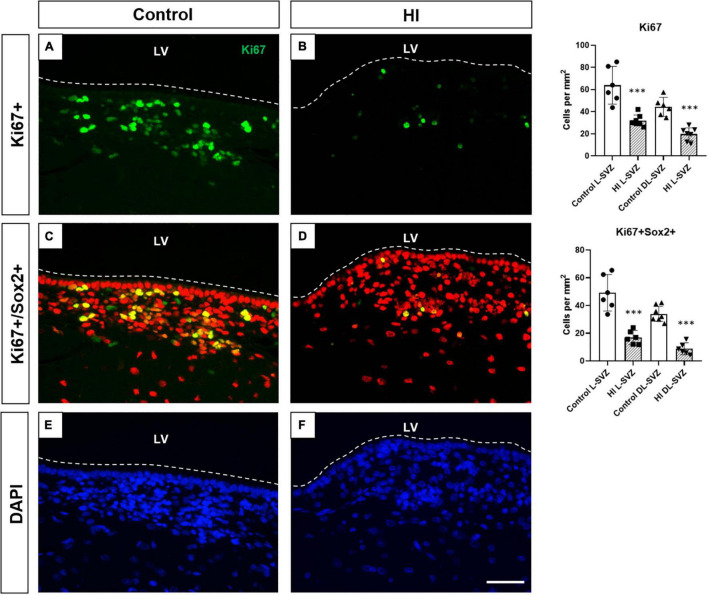
Fluorescent microphotographs of Ki67 [cell proliferation, **(A,B)**], Sox2/Ki67 [neural stem/progenitor cell proliferation, **(C,D)**], and DAPI [nuclei, **(E,F)**] immune-stained samples from control and HI piglets. Ki67 positive cells (green) are abundant in the control piglet **(A)**, whereas HI **(B)** significantly reduced its counts by half in L-SVZ and DL-SVZ (both ^***^p < 0.001). Double Sox2 (red) + /Ki67 (green) + cells are observed in control animals; again, HI reduced neural stem/progenitor cell counts in L-SVZ and DL-SVZ (both ^***^p < 0.001). DAPI immunostaining reveals total nuclei. LV, lateral ventricle; SVZ, subventricular zone; CDT, caudate nucleus; PvWM, periventricular white matter. Original magnification 400×. Scale bar: 100 μm.

Ki67-positive cells were present as individual or small clusters of cells in the SVZ of both control and HI piglets, extending several cells thick from the ependymal layer along the DL-SVZ and L-SVZ edges of the lateral ventricle (Figure 6A). Control animals showed slightly higher values of Ki67 + cells in the L-SVZ (63.89 ± 19.01 cells per mm^2^) than in the DL-SVZ (44.13 ± 9.33 cells per mm^2^). HI reduced cell proliferation by half in both L-SVZ (31.94 ± 6.98 cells per mm^2^; *p* < 0.001 vs. control) and DL-SVZ (19.44 ± 8.65 cells per mm^2^; *p* < 0.001 vs. Control) regions (Figure 6, upper graph).

Consistent with this finding, we also observed a significant decrease in the number of Sox2 + Ki67 + cells in the same regions after HI. Neural stem/progenitor cells (Sox2 +) appeared in control (Figure 6C) and HI (Figure 6D) piglets, with more presence in the L-SVZ. Its proliferation, determined by Sox2 and Ki67 double labeling, was also affected after HI, with a significant decrease in the number of Sox2 + Ki67 + cells in both L-SVZ (*p* < 0.0001 vs. control) and DL-SVZ (*p* < 0.0001 vs. control) subareas (Figure 6, lower graph).

### Neuroblast Chain Area and Doublecortin Staining Are Reduced After Hypoxia-Ischemia

**FIGURE 7:**
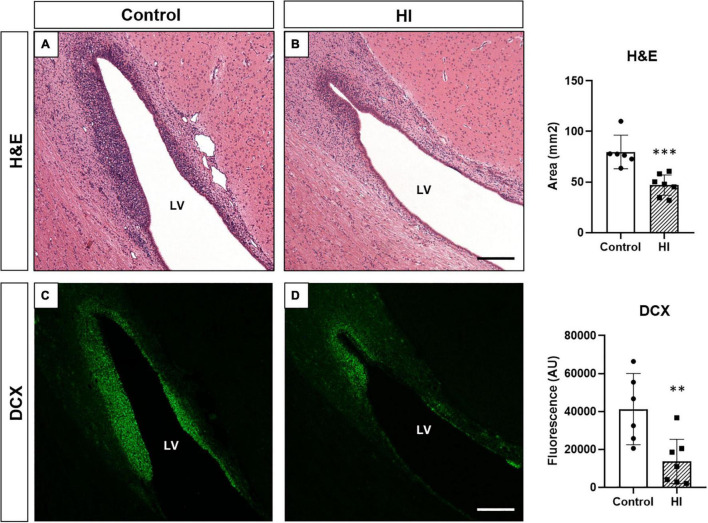
Neuroblast evaluation through hematoxylin and eosin staining [H&E, **(A,B)**] and doublecortin immunohistochemistry [DCX, **(C,D)**] from control and HI piglets. The abundant presence of neuroblasts in control animals **(A,C)** is reduced after HI **(B,D)**. Graphs: the average neuroblast chains area (^***^p < 0.001) and fluorescent intensity of DCX expression (^**^p < 0.01) in the SVZ was significantly reduced after HI. LV, lateral ventricle; H&E, hematoxylin and eosin staining; DCX, doublecortin; AU, arbitrary units Original magnification 100×. Scale bar: 500 μm.

Control animals showed an abundance of neuroblast chains (assessed by H&E staining; Figures 7A,B) and DCX + neuroblasts (immunofluorescence; Figures 7C,D). Both techniques gave us a strong positive correlation between the area of neuroblast chains and DCX immunofluorescence (*R*^2^ = 0.6447, *p* < 0.0001). HI displayed a significant reduction in the area of neuroblast chains (H&E, *p* = 0.0003) and DCX immunofluorescence (*p* = 0.0044) compared with control animals (Figure 7, graph).

## Discussion

In this study, hypoxic-ischemic piglets showed a decrease in cellularity in the SVZ independent of cell death at 48 h. Furthermore, HI decreased the length of GFAP + processes, the neuroblast chains area, DCX immunoreactivity, and the number of Ki67 + and Ki67 + Sox2 + cells, thus suggesting that, early after the HI event, a reduction in both cell proliferation and neurogenesis occurs.

Cellular and tissue damage triggered after HI can be generalized and affect the neurogenic niches present in the central nervous system. Levison et al. (6) described that moderate-to-severe HI reduced 20% of the total cells from the SVZ in rodents, so we first wanted to explore if HI could reduce the cellularity in the SVZ of the newborn piglet. The porcine SVZ is structurally similar to its human counterpart, made up of a crowd of small cells closely situated to each other, adjacent to the ependyma of the lateral ventricles and extending toward the inner brain parenchyma. In our model, HI reduced the number of cells in the piglet SVZ without increasing cell death, as the quantification of necrotic or apoptotic profiles was minimal or absent, later confirmed by the rare presence of TUNEL-positive cells. In the same manner, Morton et al. (18) found no differences in cell death within the SVZ in hypoxic brains using a porcine model of hypoxia-only injury, thus indicating that cell death is not a major underlying mechanism in the reduction of cellularity in the piglet SVZ.

Similarly, the rodent SVZ became swollen during recovery from perinatal HI (as seen here), but few of those cells died (7, 23), with cell death values of approximately 0.3% of the total SVZ cells. The same authors also evaluated caspase 3 activation, describing no differences in the percentage of active caspase-3-positive cells in either the ipsilateral or contralateral SVZs after HI in rodents (7, 23). In our work, control animals showed abundant immunoreactivity for caspase-3 in the SVZ neurogenic niche, but HI did not affect the cell counts. Indeed, caspase-3 positive nuclei were observed in non-pyknotic cells, so we cannot rule out a possible non-apoptotic role of this enzyme (24, 25) in the piglet SVZ. Despite the most recognized role of caspase-3, i.e., its capacity of inducing DNA fragmentation, its activation modulates a number of biological processes that do not cause cell death, including dendritic pruning, cell differentiation, and cell proliferation (26, 27).

Whereas the piglet SVZ could be a brain region resilient to HI-induced cell death, SVZ-surrounding regions like the periventricular white matter and caudate nucleus appeared vulnerable to HI. As previously reported by our group in piglets (16) and by other authors in rodents (23), in this study we show cellular degenerative changes in both brain regions at 48 h after HI, with low counts of morphologically well-preserved cells and high values of apoptotic and necrotic profiles, confirmed with immunohistochemical techniques. Unlike “mature tissues” like the caudate nucleus or periventricular white matter, those containing neuronal precursors like the SVZ have their particular architecture, functions, and fates (12). Together with the presence of large blood vessels affording rapid reperfusion after HI (28), neurogenic tissues often have lower oxygen levels compared with others, and their cells considerably rely upon anaerobic respiration (29), thus providing resistance to hypoxic phenomena. *In vitro* studies support the evidence for strong homeostatic controls over SVZ cells (30) and their resistance to pro-death stimuli after brain injury (23), especially when compared with the vulnerability of mature cells located in adjacent gray and white matter brain regions. These special features of SVZ cells could be partially attributed to their high levels of cytoplasmic glycogen (31) and antiapoptotic proteins (32), which could act as substrate during HI and ameliorate cell death cascades, respectively.

If cell death was not a prominent mechanism in the piglet SVZ after HI, we wondered if the reduction in SVZ cellularity could be related to a decrease in its neurogenic potential. In the SVZ, neurogenesis originates from a primary progenitor with morphological and functional characteristics of a glial cell, which expresses glial fibrillary acidic protein (GFAP) (33). GFAP was used as a surrogate for radial glia, and these GFAP + cells appeared in control animals showing a triangular shape and exhibiting long and thin processes toward the brain parenchyma. As radial glia can give rise to neural stem cells, among other cell types, we wanted to assess the neurogenic activity close to the ventricular wall, showing that HI significantly reduced the average length of the processes of GFAP + radial-glia like cells when compared with control animals, an observation also described in piglets submitted to hypoxia only (18).

Neural stem cells give rise to type 2 progenitors that undergo rapid proliferation responsible for most of the expansion of the pool of newly generated cells. The piglet SVZ exhibits high postnatal cell proliferation potency even at 6–7 or 32 weeks of age (34). This capacity was also observed in our samples from control animals, showing noticeable Ki67 + cell counts in both L- and DL-SVZ areas. However, HI induced a significant reduction in cell proliferation in the SVZ of animals post-HI, whose counts diminished by half compared with control. Consistent with this finding, we also found a reduction in the number of Sox2 + Ki67 + cells. As stated in the introduction, previous works in rodents have described that HI can either decrease (6, 7) or increase the regenerative capacity of the SVZ (9, 11). Later works suggested a possible sequential pattern in which asphyxia decreased SVZ cell proliferation at 24 h after HI, followed by an increase in cell division 7 days after the injury in the neonatal rat (35). The other neurogenic niche, the hippocampal subgranular zone, displayed a similar behavior after HI, presenting an initial decrease in cell proliferation continued by a subsequent increase after the injury (36). As a limitation of this work, we assessed only the short-term effects of HI in the neonatal piglet SVZ: the long-term effects are unknown, including a potential increase in cell proliferation or in neurogenesis in the SVZ after the initial decrease observed in this work.

In this study, the relative high abundance of neuroblasts revealed by H&E and DCX immunostaining in the SVZ of control animals was drastically reduced after HI. DCX is considered a marker of neuronal lineage/migrating neuroblasts. Its expression is thought to be specific for newly generated neurons, since nearly all DCX-positive cells express early neuronal antigens but lack antigens specific for glial, undifferentiated, or apoptotic cells (22, 37). The described biphasic pattern has also been described for DCX after traumatic brain injury: normothermic animals showed a reduction in DCX at 72 h after the injury, with subsequent recovery to their basal values at 7 days (38).

Altogether, these results suggest that neurogenesis is reduced early after neonatal HI in the SVZ of the postnatal piglet, when HI-induced cell death is still ongoing in certain regions of the brain, which could compromise the replacement of the lost neurons and the achievement of global repair.

## Data Availability Statement

The raw data supporting the conclusions of this article will be made available by the authors, without undue reservation.

## Ethics Statement

The animal study was reviewed and approved by Animal Care and Use Committee of University College London Biological Services and Institute of Neurology.

## Author Contributions

DA-A, XG, PG, and NR contributed to the acquisition, analysis, and interpretation of data. DA-A contributed to the conception and design, analysis, and interpretation of data. DA-A and NR wrote the article. All authors revised the original manuscript and agreed on its contents.

## Conflict of Interest

The authors declare that the research was conducted in the absence of any commercial or financial relationships that could be construed as a potential conflict of interest.

## Publisher’s Note

All claims expressed in this article are solely those of the authors and do not necessarily represent those of their affiliated organizations, or those of the publisher, the editors and the reviewers. Any product that may be evaluated in this article, or claim that may be made by its manufacturer, is not guaranteed or endorsed by the publisher.
