# Supplementary figures and images for: Neurogenesis Is Reduced at 48 h in the Subventricular Zone Independent of Cell Death in a Piglet Model of Perinatal Hypoxia-Ischemia
Source: Front Pediatr. 2022 Apr 28;10:793189. doi: 10.3389/fped.2022.793189 (PMC9106110; doi:10.3389/fped.2022.793189)

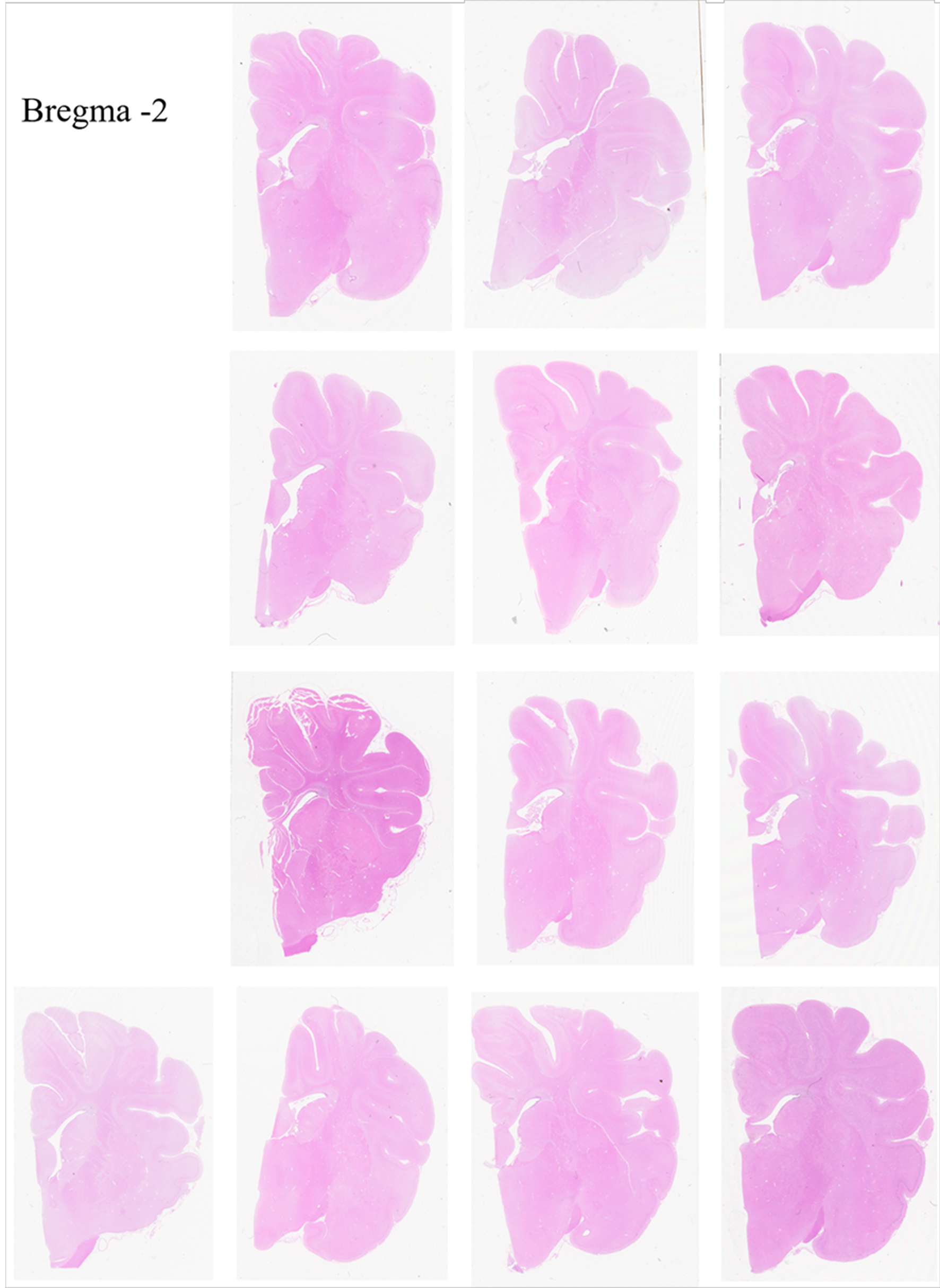

Supplement: Supplementary file 1 [file Image_1.TIF]

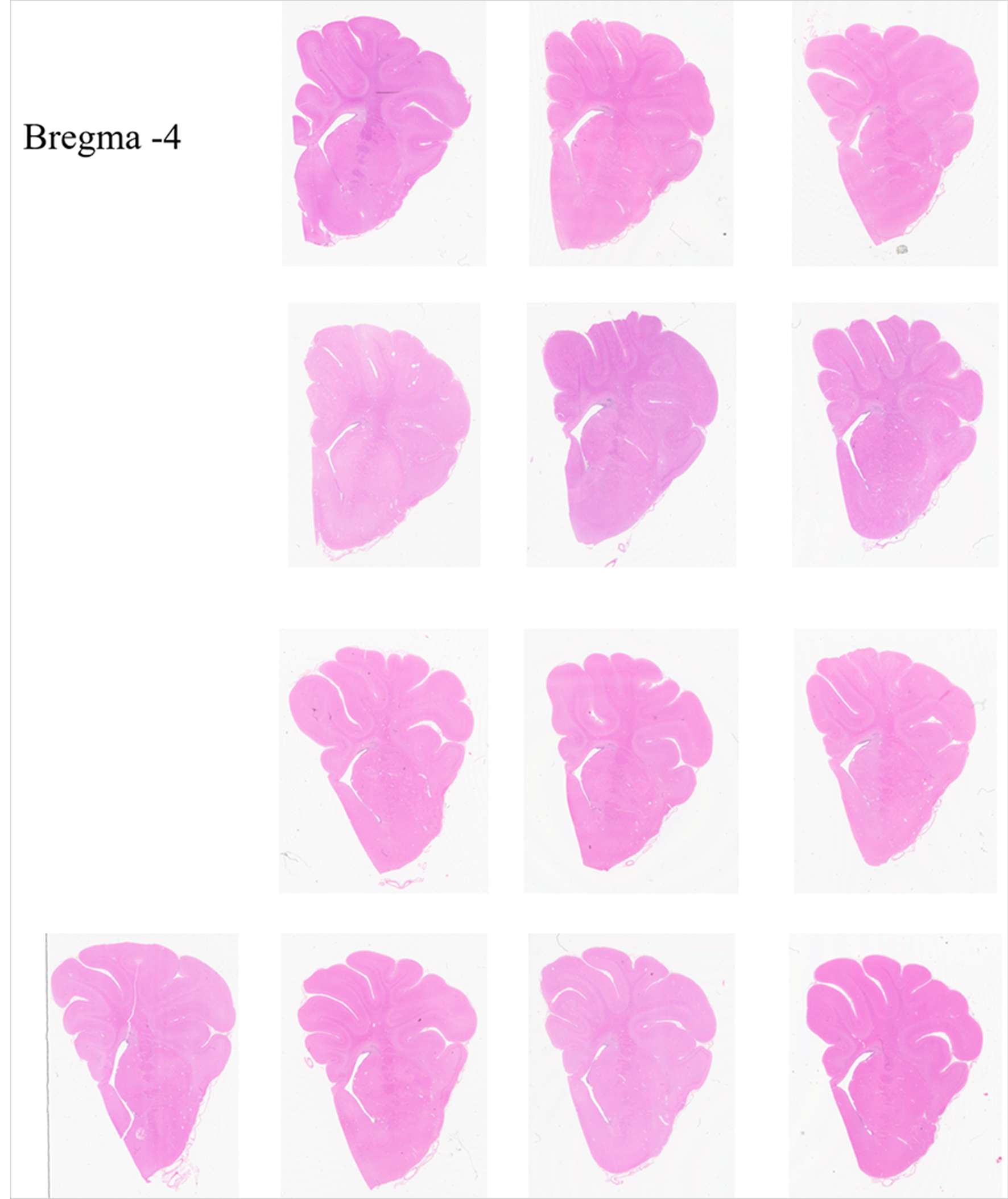

Supplement: Supplementary file 2 [file Image_2.TIF]

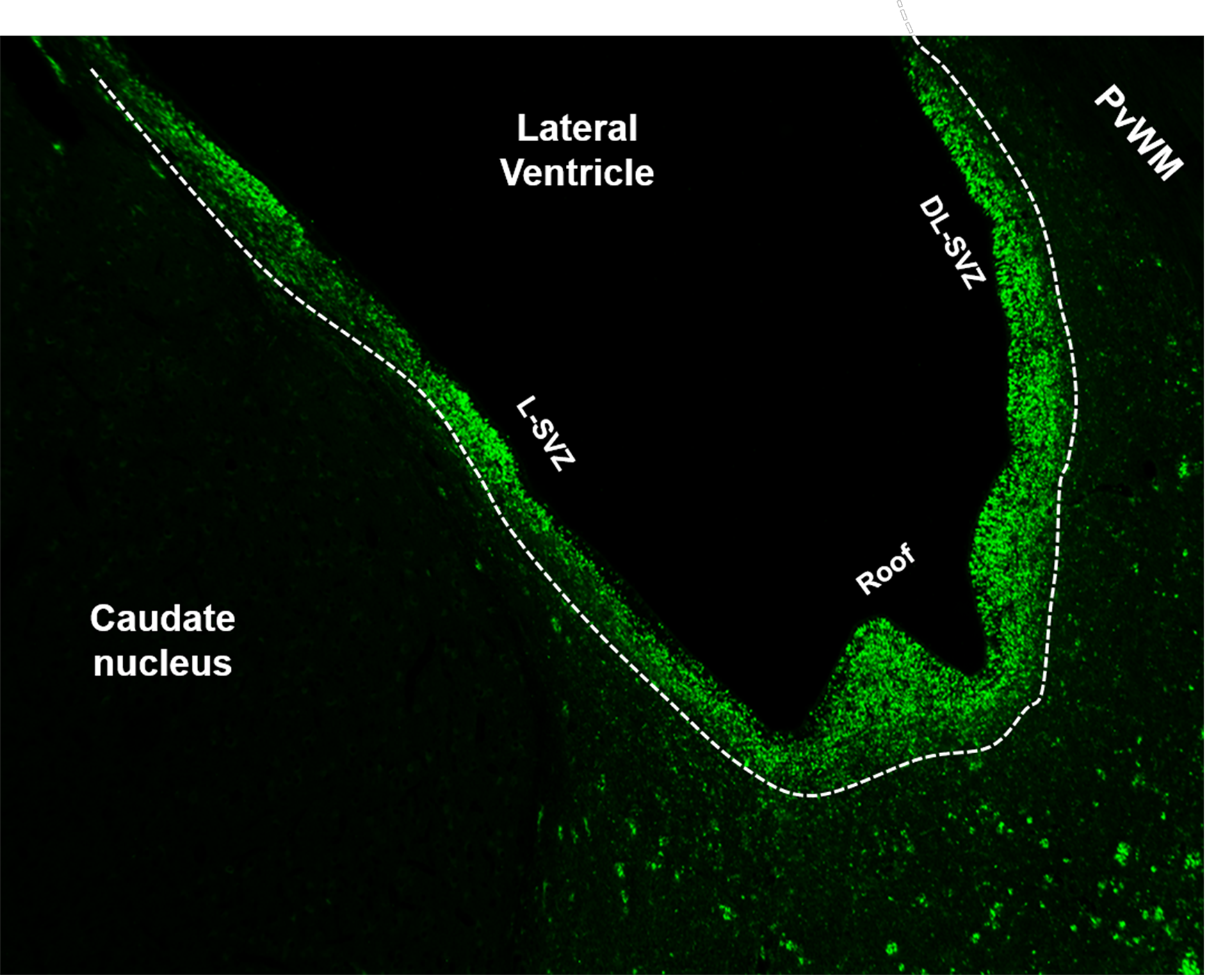

Supplement: Supplementary file 3 [file Image_3.TIF]

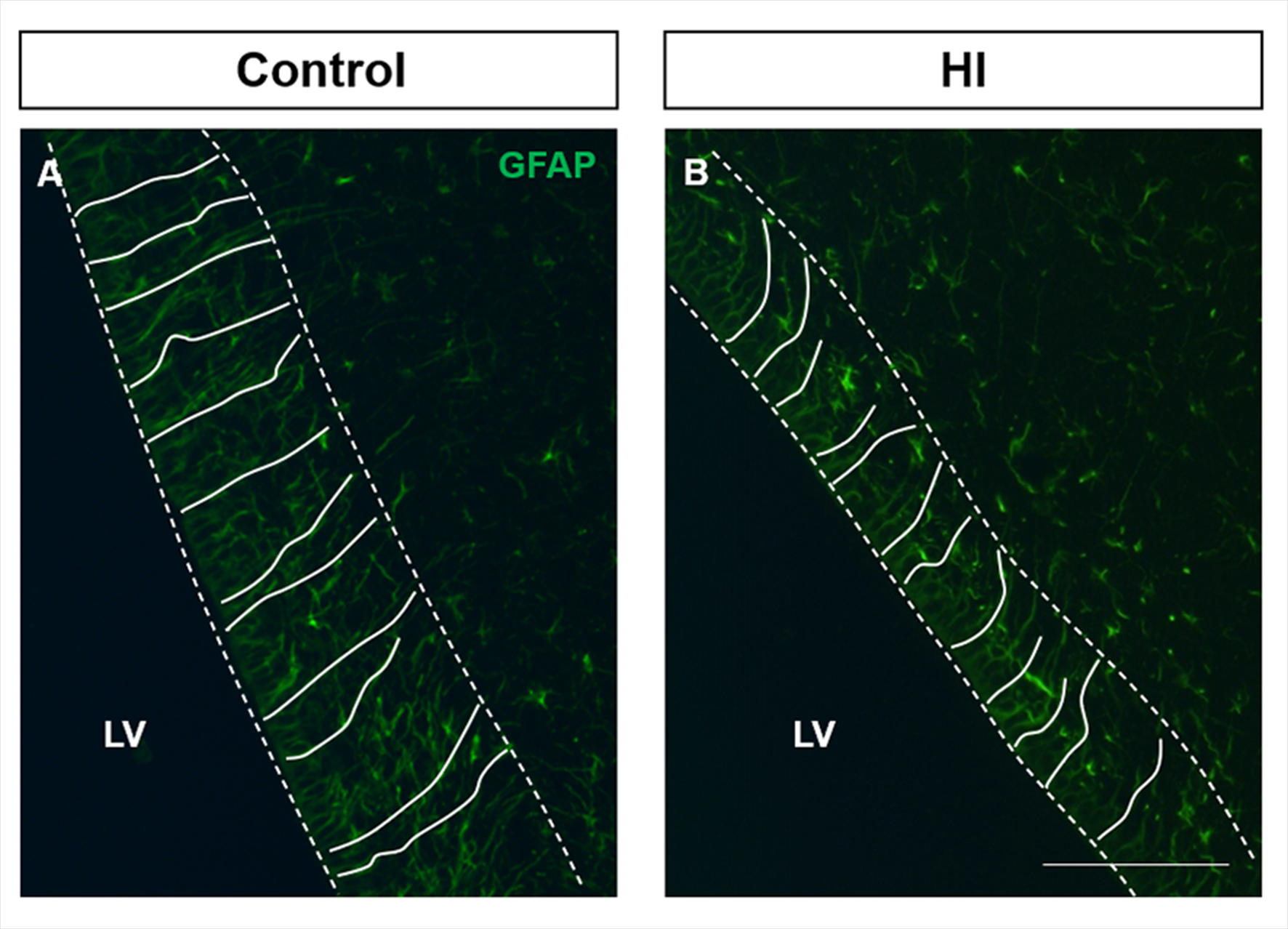

Supplement: Supplementary file 4 [file Image_4.TIF]
